# A Survey on Satisfaction of Type 2 Diabetes Patients with Different Demographic Variables to Medical Services

**DOI:** 10.3390/ijerph16071142

**Published:** 2019-03-29

**Authors:** Jui-che Tu, Yi-Lin Lee, Feng-Jung Nien

**Affiliations:** 1 Graduate School of Design, National Yunlin University of Science & Technology, Yunlin 64002, Taiwan; tujc@yuntech.edu.tw; 2 Subdivision of Endocrinology and Metabolism, Department of Internal Medicine, National Taiwan University Hospital Yun-Lin Branch, Yunlin 64002, Taiwan; fj.nien@gmail.com

**Keywords:** demographic variable, diabetes mellitus type 2, satisfaction survey

## Abstract

Taking Type 2 diabetes as the research object, and through questionnaire interviews, this study sought to determine the degree of satisfaction of patients with different attributes with medical services according to the distribution of demographic variables. Finally, the statistical results were taken as the reference basis for medical personnel to provide care to patients. Regarding the questionnaire survey, the questionnaire items were designed through face-to-face interviews aiming at their medical treatment process, thus, patients could truly reflect their feelings. This study used the SPSS statistical software (IBM, Armonk, New York, NY, USA) for analysis, and the results show that: (1) Patients of different genders had different degrees of satisfaction with medical services. (2) The difference in age, monthly disposable income, occupational category, and education level had no significant effect on service satisfaction. (3) The research subjects were all on the high side regarding their satisfaction with the service provided by medical facilities. This study is a pilot study, and it is hoped it will be used as a guideline for improving patient care quality in the future, thus, reducing the occurrence of diabetic complications through better medical care. The long-term goal is to continuously improve care and medical service quality, thus, reducing the waste of medical resources.

## 1. Introduction

With the progress of medical science, technology, and the economy, coupled with changes in eating habits and static lifestyle, diabetes has become one of the most important global public health issues in the 21st century. According to World Health Organization statistics [1], there were 410 million adults worldwide suffering from diabetes in 2016, the global diabetes prevalence rate will double from 4.7% to 8.5%, and the number of patients worldwide is expected to increase to 592 million by 2035, and it is estimated that the number of adults suffering from diabetes will rise to over 640 million by 2040. Most of them are Type 2 diabetes patients. The causes of the disease are not only congenital genetic effects, but also lifestyle [2], which can be controlled by early diagnosis, active treatment, and lifestyle changes [3]. Patients with diabetes have many needs, such as diet, medical behavior, drug therapy, and related knowledge [4]. Relevant diabetes prevention and control organizations also suggest that care teams should consider the patients’ feelings and satisfaction with medical services [5].

Ever since Taiwan’s National Health Insurance was implemented in 1995, there have been significant increases in medical resources, medical institutions and personnel, and medical-care seeking behaviors of the general public. In addition to the National Health Insurance, there are also two other completely different healthcare systems in Taiwan: traditional Chinese medicine and Western medicine. There are also folk therapies and a large number of pharmacies in neighborhoods. It is a common phenomenon that the various healthcare systems may replace one another, and as a result the Taiwanese people have their own opinions on disease perception, interpretation, and medical-care seeking behaviors. Due to the unique medical environment in Taiwan, it is impossible to interpret the general public’s behavior patterns entirely from the perspective of Western studies. In general, Western studies use a multivariate model, such as multiple medical-care seeking, family as a unit, etc. However, residents in different areas of Taiwan have developed different medical-care seeking behaviors according to the differences in their environment and socioeconomic status [6]. All in all, although there are sufficient medical resources in Taiwan, an urban-rural gap still exists. Taking the case in this study for example, the statistical data from the Taiwan Medical Association show in 2018 that there were approximately 45 physicians per 10,000 residents in the capital, Taipei City. However, Yunlin County has only 12.6 physicians per 10,000 residents. The data from the Ministry of the Interior also presents that the aging index in Yunlin County is 141.87, ranking second in Taiwan. In other words, Yunlin County is a relatively aging county. Past studies have also showed that living area affects the health behaviors of the elderly [7]. Compared with the general public living in cities, those living in aging areas engage in unhealthy behaviors more frequently, and their level of control over social and family resources is also lower [8]. Therefore, investigations associated with the medical care behaviors of the general public in specific aging areas may generate important research results. According to the literature, residents living in areas of population aging most frequently experience one or more chronic diseases [9]. The statistical data from the Public Health Bureau, in Yunlin County notes among the major causes of death in the county that diabetes ranks 3rd, suggesting that diabetes is one of the leading chronic diseases.

Based on the above, this study chose Yunlin as the research case due to its unique regional differences. Compared with other counties/cities in Taiwan, Yunlin features both population aging and a significant gap in medical resources, resulting in unique research values of the medical-care seeking behaviors of its residents. Moreover, relevant studies have indicated that differences in gender, age, marital status, educational level, and income lead to different perceptions of consultation about professional medical knowledge and use of medical services and further affect the subsequent medical-care seeking behaviors [10,11,12]. In other words, these demographic variables all affect patients’ medical-care behaviors. Therefore, it is necessary to conduct studies to investigate whether such differences among the general public lead to varying satisfaction with medical care services. The research conclusions herein are provided as improvement strategies for the existing healthcare service system. The research results may also help better understand patients’ distribution status and medical-care seeking habits so as to further achieve the objective of improving patients’ accurate medical-care seeking behaviors.

The purpose of this study is to conduct a demographic survey to explore the relationship between lifestyle and the distribution of patients with Type 2 diabetes, and to investigate patients’ visiting behaviors according to their degree of satisfaction with hospital service. The National Taiwan University Hospital, Yunlin Branch, was taken as the research field to explore the distribution of the population variables of patients with Type 2 diabetes, and whether their degree of satisfaction has any influence on visiting behavior through questionnaire survey. 

The specific objectives of this study are as follows:

1. To understand the actual feeling and satisfaction of diabetic patients regarding the environment of the medical field and the service of medical staff.
2. The results of this study can actually reflect the real feelings of patients. In addition to providing medical field care advice, the results can provide medical personnel with relevant reference basis for the demographic variable of patients.

Although the cases selected in this study have regional limitations, they are still related to relevant situations, thus, they can provide comparison in the future when faced with relevant fields and situations. Although the cases cannot directly predict solutions or guide actions, they can put forward relevant problems through comparative analysis to compare similarities and differences in different situations.

## 2. Materials and Methods

This study used purposive sampling to collect cases from internal medicine wards and the diabetes health education center of a regional teaching hospital. This study collected 223 valid questionnaires from the Type 2 diabetes patients in the National Taiwan University Hospital, Yunlin Branch. The research questionnaire includes two parts. Part one covers structural questions that collect the respondents’ basic demographic data, with the aim to perform a subsequent analysis on whether differences in patients’ demographic characteristics lead to varying satisfaction with healthcare services. This study also investigates whether the satisfaction experienced by patients after receiving healthcare services affects their subsequent medical-care seeking behaviors. Therefore, part two of this questionnaire helps examine the satisfaction experienced by patients after seeking treatments in hospital and receiving healthcare services, in order to understand whether the demographic variables of respondents affect their satisfaction with healthcare services.

The research period cover June to September in 2018. Basic demographic data and satisfaction survey of hospital services were collected through structured questionnaires. 

According to the literature, the following factors must be taken into consideration in studies on patients’ medical-case seeking behavior patterns:

(1) Care quality (medical technology quality and service quality) [13,14].
(2) Medical conveniences (distance, convenient transportation, acquaintance with hospital staff, etc.) [15,16].
(3) Hospital conditions (hardware facilities, quality, skills, and reputation of the hospital) [17].
(4) Required medical expenses [18].

For the required medical expenses mentioned in Item 4 above in the past literature, in compliance with Articles 43 and 47 of the National Health Insurance Act in Taiwan, the insured must only co-pay for their personal medical expenses. Therefore, the required medical expenses of medical visits to general public hospitals by the general public in Taiwan are the same, and patients only have to co-pay the expenses of their outpatient visits and medications. Therefore, this study does not include the required medical expenses in the questionnaire items and instead focuses on the services provided by hospitals.

The medical services provided by the medical facilities include: the service attitude of doctors and medical teams (including the registration staff, volunteers, personnel to price prescriptions and deliver drugs, senior nurses, and health educators), waiting time of patients, hardware provided by medical facilities (e.g., public service facilities, facilities for people with disabilities, and parking facilities in hospitals ), and software (e.g., moving lines, indicators, health education publicity system, related service App, and online service system). The questionnaire design of this study takes medical service satisfaction as a framework. According to the original medical services provided in the research field, the evaluation of patients’ satisfaction with medical services are divided into three major dimensions: structure, process, and results [19]. Structure refers to the services provided by the hospital, such as the hospital environment and public facilities; process refers to the services provided by medical personnel in the process, such as medical team, prescription pricing, drug collection, registration, and other services; the dimension of results: the understanding and satisfaction of the patients with the return visit mechanism via the questionnaire survey according to the behavior of patients with diabetes seeking medical treatment. The above three dimensions constitute the questionnaire of this study. The first part of the questionnaire is a basic background survey in demographic variables. A Likert five-point scale was adopted by the questionnaire to measure the interviewees in terms of the various measures of the medical treatment process, with Very Satisfied: 5 points; Satisfied: 4 points; No comment: 3 points; Dissatisfied: 2 points; Very dissatisfied: 1 point. 

The conditions for acceptance into this study were: a patient who been confirmed by a doctor’s diagnosis to be a Type 2 diabetes patient, who is literate, conscious, and can communicate with others; while the exclusion conditions are that the patient has a cognitive impairment, cannot communicate with language, is illiterate or has a mental illness and cannot fully answer the content of the questionnaire. All questionnaires were counted by the anonymous method, and 223 questionnaires were collected, all of which were valid questionnaires, for a valid recovery rate of 100%. 

The data analysis method used was the Statistical Product and Service Solutions (SPSS) version 20.0 software (IBM, Armonk, New York, NY, USA). The analysis methods were narrative statistical analysis and one-way analysis of variance.

Descriptive statistical analysis was used to describe the characteristics of the sample, as well as the average value, standard deviation, percentage, and frequency distribution of various variables. The main purpose is to present the attributes and distribution status of the research target object to show the general situation of the existing medical service users. One-way analysis of variance is suitable for testing the difference of measurement scores between more than three different independent groups. In this research questionnaire, demographic variables (age, gender, education level, monthly income, occupation) are included in the questionnaire items, and analysis of variance was used to test whether there are differences in the satisfaction of service attitude of the medical teams, including waiting time and hardware and software services, due to different demographic variables. The demographic variables in Part 1 of the questionnaire are shown in Table 1. 

The content of the satisfaction dimension in Part 2 of the questionnaire is shown in Table 2. Taking medical users as the starting point, this survey focused on satisfaction with the outpatient treatment process.

## 3. Results

The sample data of this study were analyzed and counted by the SPSS statistical software. The main statistical methods included descriptive statistics and one-way analysis of variance. The analysis process is as follows. Part one is narrative statistics.

### 3.1. The First Part of the Questionnaire, Demographic Variables 

This study collected 223 valid questionnaires from the Type 2 diabetes patients in the National Taiwan University Hospital, Yunlin Branch. The demographic variables of the patients include gender, age, education level, occupation category, and monthly disposable income. Table 3 is the gender number of times allocation, Table 4 is the age number of times allocation, and Table 5 is the gender and age cross tabulation.

According to the statistical results of this research questionnaire, the proportion of male and female is 45.7% and 54.3%, respectively, indicating that the majority of the research objects are women.

Regarding the age distribution of the subjects in this study, four people were between 20 to 30 years old (1.8%), 32 people were aged 30–50 (14.3%), 52 people were aged 50–60 (23.3%), and 135 people were aged over 60 (60.5%). 

As shown in the cross analysis table, regardless of the gender of the interviewee, the population aged over 60 accounts for the majority.

The following Table 6 is the education level number of times allocation, and Table 7 is the gender and education level cross tabulation.

There are 129 subjects with an educational level below primary school (7.8%), 21 subjects with junior high school-level edication (9.4%), 38 subjects with a senior high school (vocational high school) level (17%), 11 junior college education subjects (4.9%), 16 subjects with a university degree (7.2%), and eight subjects with graduate school education (3.6%).

As shown in the cross analysis table, regardless of gender of the interviewee, the educational level distribution was mostly below primary school.

The following Table 8 is the occupation number of times allocation, and Table 9 is the gender and career cross tabulation.

Regarding the occupation of the subjects of this study, there were two students (0.9%), nine soldiers, public servants, and teachers (4%), 11 in industrial manufacturing industries (4.9%), 17 in business service industries (7.6%), 32 in agriculture, forestry, fishery, animal husbandry, and mining industries (14.3%), 10 freelancers (4.5%), and 142 other (including retirees) (63.7).

As shown in the cross analysis table, regardless of gender of the interviewee, the occupation of the subjects of this study was mostly retired.

The following Table 10 is the monthly disposable income number of times allocation, and Table 11 is the gender and monthly disposable income cross tabulation.

New Taiwan dollar (NTD) is the official currency units in Taiwan. Regarding the monthly disposable income of the subjects, 118 subjects had below NTD 10,000 (52.9%), 50 has NTD 10,001–30,000 (22.4%), 36 has NTD 30,001–50,000 (16.1%), 13 has NTD 50,001–100,000 (5.8%), and six had NTD 10,0001 or above (2.7%). 

As shown in the cross analysis table, regardless of gender, the monthly disposable income was mostly NTD 10,000 or less. According to the demographic variables, the sample statistics of patients with Type 2 diabetes show that females were more numerous than males, and the population over 60 years old was the majority. The distribution of educational level was mostly below primary school, and occupation is mainly other (including retirees). The monthly disposable income was mostly below NTD 10,000.

### 3.2. The Second Part of the Questionnaire is the Satisfaction Survey 

The second part is the satisfaction survey. First, the questionnaire items are listed in the table. According to literature, the evaluation of medical service satisfaction can be divided into three major dimensions: structure, process, and results:

(1) Structure: the service provided by the hospital; this dimension was named the service satisfaction of the medical structure.
(2) Process: the service provided by medical personnel in the process; this dimension was named as medical process service satisfaction.
(3) Results: According to the patient’s behavior of seeking medical treatment, the questionnaire was used to inquire about the status and satisfaction of the return visit and the follow-up medication mechanism; this dimension was named as the satisfaction of medical service results.

According to the literature, all items of the questionnaire were integrated into the above three dimensions, and then, the scores of each dimension were averaged and integrated into a single variable for subsequent statistical analysis. The research dimension scale is shown as follow Table 12, Table 13 and Table 14: 

### 3.3. Differences in Satisfaction of Population Variables 

In this part, the average score of satisfaction of the three dimensions is calculated by using the demographic variables, respectively, to learn whether the scores of each dimension are high or low for different demographic variables. Table 15 shows the result of using gender to calculate the average score of satisfaction of the three dimensions.

According to the table, the scores of both genders in the dimensions of medical structure, process, and results of the questionnaire are even. The highest score is women’s satisfaction with medical structure service, with an average score of 3.74, and the lowest is their satisfaction with medical result service, with a score of 3.6. The highest score for men is the same as that for medical structure service, with an average score of 3.75, and the lowest is the same as that for medical result service, with a score of 3.59. 

The following Table 16 shows the result of using age to calculate the average score of satisfaction of the three dimensions.

The scores of the subjects of different ages in all aspects of the questionnaire are even, with the highest score of medical process service satisfaction being 3.79, ranging from 20 to 30 years old, and the lowest score being 3.71, ranging from 50 to 60 years old. The highest score for the satisfaction degree of medical process service is 3.91, which falls in the range of 20–30 years old, and the lowest is 3.63, which falls in the range of 50–60 years old. The highest score for the satisfaction degree of medical service results is 3.78, falling in the 20–30 age range, and the lowest score is 3.56, falling in the 50–60 age range. 

The scores of the various educational levels in all aspects of the questionnaire are even, with the highest score of 3.81 for medical process service satisfaction, falling within the junior high school range, and the lowest score of 3.63, falling within the university range. The highest score of the satisfaction degree of medical process service is 3.76, which falls in the university section, and the lowest is 3.63, which falls in the junior college section. The highest score for the satisfaction degree of medical service results is 3.67, falling in the university range, and the lowest is 3.57, falling in the high school (vocational high school) range.

The following Table 17 shows the result of using education level to calculate the average score of satisfaction of the three dimensions.

The following Table 18 shows the result of using occupation to calculate the average score of satisfaction of the three dimensions.

The scores of the different occupations in all aspects of the questionnaire are even, with the highest score of 3.77 for medical process service satisfaction, falling within the range of agriculture, forestry, fishing, animal husbandry, and mining industries, and the lowest score of 3.45, falling within the section of students. The highest satisfaction degree of medical process service is 3.81, which falls in the section of soldiers, public servants, and teachers, and the lowest is 3.47, which falls in the section of students. The highest score for the satisfaction degree of medical service results is 3.77, falling within the range of soldiers, public servants, and teachers, and the lowest is 3.52, falling within the industrial manufacturing range.

The following Table 19 shows the result of using monthly disposable income to calculate the average score of satisfaction of the three dimensions.

The scores of the different occupational categories are even in all aspects of the questionnaire, with the highest score of 3.81 for medical process service satisfaction, falling within the range of monthly disposable income of NTD 100,001 or above, and the lowest score of 3.62, falling within the range of monthly disposable income of 50,001–100,000. The highest score for the satisfaction degree of medical process service is 3.85, which falls in the range of monthly disposable income of NTD 100,001 or above, and the lowest is 3.62 and falls in the range of monthly disposable income of NTD 50,001–100,000. The highest score for the satisfaction degree of medical service results is 3.71, falling within the range of monthly disposable income of NTD 100,001 or above, and the lowest is 3.57, falling within the range of monthly disposable income of NTD 30,001–50,000. All the above currency units were New Taiwan Dollars.

### 3.4. One-way Analysis of Variance 

In this part, according to the questionnaire dimension scale, the data were used to test the differences in the structure, process, and results of the dimension content through statistical methods aiming at the demographic variables of the research object as the variance. The purpose is to explore whether there are significant differences in the perception of medical service satisfaction among groups of different attributes. Table 20 shows the narrative statistics analysis of gender and scale dimension variables, and Table 21 is the result of analysis of variance.

From the table, it can be seen that there are significant differences between males and females in the degree of satisfaction with medical process service, where the average score of males is 3.71 and that of females is 3.64. Male patients are more satisfied with the service process provided by medical institutions, as compared with female patients. 

The following Table 22 is analysis of variance between age and scale dimension.

It can be seen from the table that there is no significant difference in the satisfaction scores of patients for all dimensions, regardless of whether they are under 20 years old, 20–30 years old, 30–50 years old, 50–60 years old, or over 60 years old. 

The following Table 23 is analysis of variance between educational level and scale dimension.

According to the statistical results, there is no significant difference in satisfaction scores for all service components in the hospital, regardless of whether the educational level was primary school or below, junior high school, senior high school (vocational high school), junior college, university, and graduate school or above. 

The following Table 24 is analysis of variance between occupational category and scale dimension.

From the table, it can be seen that the patients’ satisfaction scores for all service components in the hospital are not significantly different, regardless of their occupation: students, soldiers, public servants and teachers, industrial manufacturing, business service industry, agriculture, forestry, fishing, animal husbandry, mining, freelance, or others. 

The following Table 25 is analysis of variance between monthly disposable income and scale dimension.

From the table, it can be seen that patients’ satisfaction scores for all service components in the hospital have no significant difference, regardless of whether their disposable income is below 10,000, NTD 10,001–30,000, NTD 30,001–50,000, NTD 50,001–100,000, and above NTD 10,000.

## 4. Discussion

In this study, 223 patients from the National Taiwan University Hospital, Yunlin Branch, were selected as the research subjects, all of whom were diagnosed as having Type 2 diabetes by the attending doctors. The results show that there were no significant differences in the satisfaction scores of demographic variables, such as age, education level, occupation category, and monthly disposable income for the services provided in the medical facilities in terms of structure (hardware and environment), process, and results. However, there was one variable, gender, which showed a significant difference in satisfaction with the medical process service. Statistical results show that male patients had higher scores than female patients in the service process provided by medical institutions, that is, the service provided in the process by medical personnel (including the overall process of medical team inquiry, prescription pricing, drug collection, registration, and drug description). On the whole, the statistical data of this study shows that patients’ satisfaction with the hardware facilities in the medical facilities was high. Although there were differences in scores regarding process, the overall average score was still above 3.5 points. In the dimension of results, there was no group difference. On the whole, the statistical data of this study found that the patients were older, most of whom were over 60 years old, with lower education level, most of whom had retired, and earning less than NTD 10,000, and the average score of each dimension was above 3.5 points. It was obvious that they had a certain positive feeling of satisfaction with medical services. Although this study adopted the purposive sampling method, and was limited by the lack of research manpower and time, face-to-face and anonymous single inquiries were directly adopted as the investigation method, in order that the interviewees could truly reflect their feelings, and the statistical results are still of reference value. This study can be used as a reference for subsequent medical service personnel, who may use it as a basis and guideline for service improvement. 

## 5. Conclusions

This study explored whether groups of different demographic variables have different feelings and degrees of satisfaction with the services provided by medical facilities through a satisfaction survey. Although this study has regional limitations, and it is not possible to extend the results to all fields, the place where the study was conducted was a regional medical institution. Hence, the relevant results can still be used as control and reference for similar situations in the future. Moreover, different parts can be pointed out in different situations through comparisons and proposal of related problems. In addition, this study did not intend to survey the satisfaction of all the people with medical services, but focused on patients with Type 2 diabetes in our hospital, which aimed to break through the general questionnaire survey method, while neglecting the in-depth exploration and research of specific groups. Although the large number of samples are representative, the questionnaire, which was designed for the actual situation faced by specific objects, can still provide reliable results. The nature of this study is a pilot study. In this study, patients’ understanding of service quality is classified into the “medical result service” part of the scale dimension. It is suggested that follow-up studies can track the status of controlling patients’ glycated hemoglobin to determine whether patients’ medical cognition and service satisfaction have influence on their own disease control degree, and then, to speculate whether the level of patients’ service satisfaction will interfere with patients’ behavior and decisions in selecting medical institutions, thus, affecting disease control.

This study takes the National Taiwan University Hospital, Yunlin Branch as its research case sample. Located in Douliu City, Yunlin, Taiwan. This hospital offers a wide range of medical services. We find that patients in Yunlin exhibit differences from those patients in other counties/cities in Taiwan. The questionnaire scores also show no significant difference in satisfaction with transportation services offered by the healthcare institution. For the demographic characteristics of the patients, 60.5% are the elderly, and the educational level of 57.8% of all patients is elementary school (and under). The research results illustrate no significant difference in satisfaction with the services provided by the healthcare institution, including structures (hardware and environment), processes, and outcomes. We can see that patients’ satisfaction with the services provided by the hospital is almost the same, despite the differences in their demographic variables. However, the patients in this study are mainly the elderly with a very low educational level. For them, there is no significant difference in their satisfaction with the medical services they received. 

The data from the Department of Statistics, Ministry of Health and Welfare in Taiwan present that a mean of 44.38 patients per 100,000 with diabetes in every county/city in Taiwan passed away. For Yunlin County, this number went up to 70 patients who passed away, ranking 2nd nationwide and far exceeding the mean. Diabetes is a chronic disease, and many past clinical experiments and trials have proved in addition to receiving medical diagnosis and medication control that patients also have to receive long-term care and methods of control in order to effectively help relieve their disease condition. According to the statistical results herein, future studies are advised to use other qualitative research methods, such as in-depth interviews and field research, to investigate whether other medication, care, and life patterns of elderly patients with diabetes whose educational level is lower are affected by other factors. Such factors may result in a higher mortality rate of patients with diabetes in Yunlin compared with those in other counties/cities after they receive medical diagnosis, despite their high satisfaction with services.

## Figures and Tables

**Table 1:** Demographic variables.

Background Variable	Variable Items
Gender	Male, female
Age	Under 20, 20–30, 30–50, 50–60, over 60
Educational level	Below primary school (inclusive), above junior high school, senior high school (vocational high school), junior college, university, graduate school (inclusive) and above
Occupation	Students, soldiers, public servants and teachers, industrial manufacturing, commercial services, agriculture, forestry, fishing, animal husbandry and mining, freelance, and others
Monthly disposable income	NT$ 10,000 or less, NT $ 10,001–30,000, NT $ 30,001–50,000, NT $ 50,001–100,000, NT $ 100,001 or more

**Table 2:** Satisfaction dimension.

Dimension	Description of Content
Hospital service quality	Including satisfaction with the software and hardware, environmental facilities, and transportation services provided by the hospital
Waiting time	Satisfaction of waiting time for registration, medical treatment, drug collection, inspection, and use of hospital-provided medical system
Medical treatment	Patient‘s satisfaction with completing the complete treatment process from registration
Medication	Satisfaction with the instructions for medication and the service provided by the hospital
Return visit and other aspects	Patient‘s satisfaction with return visit date and knowledge description service provided by the hospital

**Table 3:** Gender.

Valid	Number of Times Allocation	Percentage	Effective Percentage	Cumulative Percentage
Female	121	54.3	54.3	54.3
Male	102	45.7	45.7	100.0
Total	223	100.0	100.0	

**Table 4:** Age.

Valid	Number of Times Allocation	Percentage	Effective Percentage	Cumulative Percentage
20–30 years old	4	1.8	1.8	1.8
30–50 years old	32	14.3	14.3	16.1
50–60 years old	52	23.3	23.3	39.5
Over 60 years old	135	60.5	60.5	100.0
Total	223	100.0	100.0	

**Table 5:** Gender and age cross list.

Gender *Age Cross List						
Count		20–30 Years Old	30–50 Years Old	50–60 Years Old	Over 60 Years Old	Total
Gender	Female	2	16	28	75	121
	Male	2	16	24	60	102
Total		4	32	52	135	223

* Cross tabulation.

**Table 6:** Educational level.

Valid	Number of Times Allocation	Percentage	Effective Percentage	Cumulative Percentage
Below primary school (inclusive)	129	57.8	57.8	57.8
Junior high school	21	9.4	9.4	67.3
High school (vocational high school)	38	17.0	17.0	84.3
Junior college	11	4.9	4.9	89.2
University	16	7.2	7.2	96.4
Graduate school or above	8	3.6	3.6	100.0
Total	223	100.0	100.0	

**Table 7:** Gender and educational level cross list.

Gender * (3) Educational Level Cross List								
Count		Below Primary School (Inclusive)	Junior High School	High School (Vocational High School)	Junior College	University	Graduate School or Above	Total
Gender	Female	74	14	16	6	9	2	121
	Male	55	7	22	5	7	6	102
Total		129	21	38	11	16	8	223

* Cross tabulation.

**Table 8:** Occupation.

Valid	Number of Times Allocation	Percentage	Effective Percentage	Cumulative Percentage
Student	2	0.9	0.9	0.9
Soldiers, public servants and teachers	9	4.0	4.0	4.9
Industrial manufacturing	11	4.9	4.9	9.9
Business services	17	7.6	7.6	17.5
Agriculture, forestry, fishing, animal husbandry and mining	32	14.3	14.3	31.8
Freelance	10	4.5	4.5	36.3
Other (including retirees)	142	63.7	63.7	100.0
Total	223	100.0	100.0	

**Table 9:** Gender and Career Cross List.

Gender * Career Cross List									
Count		Student	Soldiers, Public Servants and Teachers	Industrial Manufacturing	Business Services	Agriculture, Forestry, Fishing, Animal Husbandry and Mining	Freelance	Other (Including Retirees)	Total
Gender	Female	1	7	3	11	13	5	81	121
	Male	1	2	8	6	19	5	61	102
Total		2	9	11	17	32	10	142	223

* Cross tabulation.

**Table 10:** Monthly disposable income.

Valid	Number of Times Allocation	Percentage	Effective Percentage	Cumulative Percentage
10,000(inclusive) and below	118	52.9	52.9	52.9
10,001–30000	50	22.4	22.4	75.3
30,001–50000	36	16.1	16.1	91.5
50,001–100000	13	5.8	5.8	97.3
100,001(inclusive) and above	6	2.7	2.7	100.0
Total	223	100.0	100.0	

**Table 11:** Gender and monthly disposable income cross list.

Gender * Monthly Disposable Income Cross List							
Count		NTD 10,000 (Inclusive) and Below NTD 10,000 or Less	NTD 10,001 –30,000	NTD 30,001-50,000	NTD 50,001–100,000	NTD 100,001 (Inclusive) and AboveNTD 100,001 or Above	Total
Gender	Female	67	31	14	6	3	121
	Male	51	19	22	7	3	102
Total			50	36	13	6	223

* Cross tabulation.

**Table 12:** Scale of medical structure service dimension.

Question Number	Question Item
A1	The waiting room has moderate air conditioning (for cooling and heating)
A2	Facilities in waiting room (TV, seats, light, etc.)
A3	Explicit signs or billboards
A4	Activities in waiting room (TV broadcast or health education information)
A5	Cleaning of floor and toilet
A6	Hospital shuttle service
A7	Transportation convenience provided by hospital (parking lot, etc.)
A8	Visible medical facilities (wheelchairs, etc.) provided by hospitals
A9	Hospital online service system (online registration, telephone reservation system)
A10	Hospital entity service system (prescription pricing and drug collection counter)
A11	Hospital medical facilities are safe and reliable

**Table 13:** Scale of medical process service dimension.

Question Number	Question Item
B1	The length of waiting time for registration
B2	The length of waiting time for seeing a doctor
B3	The length of meeting a doctor
B4	The length of time to wait for the same day’s examination (blood drawing, x-ray, other examinations)
B5	The time to arrange examination or inspection at another time
B6	Time to wait for results of inspection report on the same day
B7	The length of time to wait for the price of the prescription
B8	The length of time to wait for the drug collection
B9	The length of time to wait for the shuttle bus
B10	The length of time to wait for using the medical system (e.g., online appointment registration and consultation status inquiry)
B11	Health Insurance Card insertion registration sequence process
B12	Counter staff’s description of the visit process
B13	Senior nurse’s statement on post-treatment process
B14	Medical service staff’s explanation on doubts about illness
B15	Interpretation of medication information by pharmacy staff
B16	Physician’s explanation of doubts about illness
B17	Health educator’ interpretation of health education information
B18	The prescription pricing and registration process is clear

**Table 14:** Satisfaction with medical service results.

Question Number	Question Item
C1	The hospital provides instructions on the medicine bag
C2	Instructions for drug use by hospital pharmacy staff
C3	Instructions from hospital pharmacy staff for prescription and drug collection
C4	Physician’s instructions for medication
C5	Physician’s instructions for prescription and drug collection
C6	Senior nurse’s instructions for medication
C7	Senior nurse’s instructions for prescription and drug collection
C8	Health educator’s instructions for medication
C9	Hospital provides medication information instructions (App or on paper, etc.)
C10	Instructions for mediation from pharmacists for drug collection in non-hospital pharmacy
C11	Instructions for prescription and drug collection from pharmacists for drug collection in non-hospital pharmacy
C12	I can clearly understand the time and date on the return visit form provided by the hospital
C13	I can get the date of appointment made by the doctor for return visit
C14	I can get the time of return visit notified by the senior nurse
C15	I can get the time of return visit notified by the health educator
C16	I can get the time of return visit through the instructions attached to the medicine bag
C17	I can get the time of return visit through the instructions of the hospital counter staff
C18	I can get the time of return visit via being reminded by my relatives
C19	I can control the time of return visit without being reminded
C20	I can exchange relevant information through patient groups
C21	I can exchange relevant information through other patients in the waiting room

**Table 15:** Gender.

Gender		Satisfaction of Medical Structure Service	Satisfaction with Medical Process Service	Satisfaction with Medical Results and Services
Female	Average value	3.7498	3.6405	3.6044
	N	121	121	120
	Standard deviation	0.29813	0.25144	0.21330
Male	Average value	3.7567	3.7157	3.5901
	N	102	102	102
	Standard deviation	0.21716	0.26026	0.20160
Total	Average value	3.7530	3.6749	3.5978
	N	223	223	222
	Standard deviation	0.26365	0.25768	0.20766

**Table 16:** Age.

Age		Satisfaction of Medical Structure Service	Satisfaction with Medical Process Service	Satisfaction with Medical Results and Services
20–30 years old	Average value	3.7955	3.9167	3.7857
	N	4	4	4
	Standard deviation	0.13636	0.22453	0.16265
30–50 years old	Average value	3.7386	3.7014	3.5883
	N	32	32	31
	Standard deviation	0.19006	0.21895	0.23700
50–60 years old	Average value	3.7185	3.6346	3.5614
	N	52	52	52
	Standard deviation	0.22141	0.24683	0.21655
Over 60 years old	Average value	3.7684	3.6770	3.6085
	N	135	135	135
	Standard deviation	0.29483	0.26843	0.19596
Total	Average value	3.7530	3.6749	3.5978
	N	223	223	222
	Standard deviation	0.26365	0.25768	0.20766

**Table 17:** Educational level.

Educational Level		Satisfaction of Medical Structure Service	Satisfaction with Medical Process Service	Satisfaction with Medical Results and Services
Below primary school	Average value	3.7541	3.6680	3.5919
	N	129	129	128
	Standard deviation	0.24541	0.26837	0.18560
Junior high school	Average value	3.8139	3.6720	3.5918
	N	21	21	21
	Standard deviation	0.14792	0.22216	0.09709
High school (vocational high school)	Average value	3.7608	3.6769	3.5752
	N	38	38	38
	Standard deviation	0.26434	0.20211	0.22995
Junior college	Average value	3.7686	3.6364	3.6190
	N	11	11	11
	Standard deviation	0.13088	0.13232	0.10859
University	Average value	3.6364	3.7604	3.6726
	N	16	16	16
	Standard deviation	0.50562	0.36273	0.42623
Graduate school or above	Average value	3.7500	3.6667	3.6369
	N	8	8	8
	Standard deviation	0.23681	0.32394	0.07171
Total	Average value	3.7530	3.6749	3.5978
	N	223	223	222
	Standard deviation	0.26365	0.25768	0.20766

**Table 18:** Occupation.

Occupation		Satisfaction of Medical Structure Service	Satisfaction with Medical Process Service	Satisfaction with Medical Results and Services
Students	Average value	3.4545	3.4722	3.7143
	N	2	2	1
	Standard deviation	0.64282	0.66782	
Soldiers, public servants and teachers	Average value	3.6465	3.8148	3.7725
	N	9	9	9
	Standard deviation	0.63220	0.41295	0.48374
Industrial manufacturing	Average value	3.7438	3.7576	3.5281
	N	11	11	11
	Standard deviation	0.16673	0.35335	0.38241
Business services	Average value	3.7647	3.6405	3.5742
	N	17	17	17
	Standard deviation	0.21575	0.22491	0.18013
Agriculture, forestry, fishing, animal husbandry and mining	Average value	3.7756	3.6597	3.5759
	N	32	32	32
	Standard deviation	0.21536	0.20142	0.11240
Freelance	Average value	3.6545	3.6000	3.6762
	N	10	10	10
	Standard deviation	0.29629	0.24117	0.07027
Other (including retirees)	Average value	3.7650	3.6753	3.5936
	N	142	142	142
	Standard deviation	0.24046	0.24804	0.18470
Total	Average value	3.7530	3.6749	3.5978
	N	223	223	222
	Standard deviation	0.26365	0.25768	0.20766

**Table 19:** Monthly Disposable Income.

Monthly Disposable Income		Satisfaction of Medical Structure Service	Satisfaction with Medical Process Service	Satisfaction with Medical Results and Services
10,000 or below	Average value	3.7581	3.6737	3.5950
	N	118	118	117
	Standard deviation	0.23956	0.26425	0.18193
NTD 10,001–30,000	Average value	3.7545	3.6556	3.5895
	N	50	50	50
	Standard deviation	0.17925	0.22023	0.13568
NTD 30,001–50,000	Average value	3.7702	3.6944	3.5714
	N	36	36	36
	Standard deviation	0.20326	0.22003	0.24467
NTD 50,001–100,000	Average value	3.6224	3.6239	3.6740
	N	13	13	13
	Standard deviation	0.55403	0.29931	0.27067
NTD 100,001 or above	Average value	3.8182	3.8519	3.7143
	N	6	6	6
	Standard deviation	0.59196	0.48390	0.57063
Total	Average value	3.7530	3.6749	3.5978
	N	223	223	222
	Standard deviation	0.26365	0.25768	0.20766

**Table 20:** Analysis of gender and scale dimension variables.

Narrative Statistics									
Questionnaire Dimension Scale		N	Average Value	Standard Deviation	Standard Error	95% Confidence Interval of Average Value		Minimum Value	Maximum Value
						Lower Limit	Upper Limit		
Satisfaction of medical structure service	Female	121	3.7498	0.29813	0.02710	3.6962	3.8035	2.55	5.00
	Male	102	3.7567	0.21716	0.02150	3.7140	3.7993	3.00	4.00
	Total	223	3.7530	0.26365	0.01766	3.7182	3.7877	2.55	5.00
Satisfaction with medical process service	Female	121	3.6405	0.25144	0.02286	3.5952	3.6858	3.00	4.78
	Male	102	3.7157	0.26026	0.02577	3.6646	3.7668	3.00	4.28
	Total	223	3.6749	0.25768	0.01726	3.6409	3.7089	3.00	4.78
Satisfaction with medical results and services	Female	120	3.6044	0.21330	0.01947	3.5658	3.6429	2.95	4.71
	Male	102	3.5901	0.20160	0.01996	3.5505	3.6297	2.48	4.00
	Total	222	3.5978	0.20766	0.01394	3.5703	3.6253	2.48	4.71

**Table 21:** Variance analysis.

Variance Analysis						
Questionnaire Dimension Scale		Sum of Squares	Variance	Mean Square	F	Significance
Satisfaction of medical structure service	Between groups	0.003	1	0.003	0.037	0.847
	Within a group	15.429	221	0.070		
	Total	15.431	222			
Satisfaction with medical process service	Between groups	0.313	1	0.313	4.793	0.030
	Within a group	14.428	221	0.065		
	Total	14.741	222			
Satisfaction with medical results and services	Between groups	0.011	1	0.011	0.259	0.611
	Within a group	9.519	220	0.043		
	Total	9.530	221			

**Table 22:** Analysis of age and scale dimension variables.

Variance Analysis						
Questionnaire Dimension Scale		Sum of Squares	Variance	Mean Square	F	Significance
Satisfaction of medical structure service	Between groups	0.107	3	0.036	0.512	0.675
	Within a group	15.324	219	0.070		
	Total	15.431	222			
Satisfaction with medical process service	Between groups	0.341	3	0.114	1.730	0.162
	Within a group	14.400	219	0.066		
	Total	14.741	222			
Satisfaction with medical results and services	Between groups	0.228	3	0.076	1.785	0.151
	Within a group	9.302	218	0.043		
	Total	9.530	221			

**Table 23:** Analysis of educational level and scale dimension variables.

Variance Analysis						
Questionnaire Dimension Scale		Sum of Squares	Variance	Mean Square	F	Significance
Satisfaction of medical structure service	Between groups	0.301	5	0.060	0.862	0.507
	Within a group	15.131	217	0.070		
	Total	15.431	222			
Satisfaction with medical process service	Between groups	0.140	5	0.028	0.417	0.836
	Within a group	14.601	217	0.067		
	Total	14.741	222			
Satisfaction with medical results and services	Between groups	0.131	5	0.026	0.604	0.697
	Within a group	9.399	216	0.044		
	Total	9.530	221			

**Table 24:** Analysis of occupational category and scale dimension variables.

Variance Analysis						
Questionnaire Dimension Scale		Sum of Squares	Variance	Mean Square	F	Significance
Satisfaction of medical structure service	Between groups	0.417	6	0.070	1.001	0.426
	Within a group	15.014	216	0.070		
	Total	15.431	222			
Satisfaction with medical process service	Between groups	0.417	6	0.070	1.048	0.395
	Within a group	14.324	216	0.066		
	Total	14.741	222			
Satisfaction with medical results and services	Between groups	0.430	6	0.072	1.695	0.124
	Within a group	9.100	215	0.042		
	Total	9.530	221			

**Table 25:** Analysis of Monthly Disposable Income and Scale Dimension Variables.

Variance Analysis						
Questionnaire Dimension Scale		Sum of Squares	Variance	Mean Square	F	Significance
Satisfaction of medical structure service	Between groups	0.261	4	0.065	0.938	0.443
	Within a group	15.170	218	0.070		
	Total	15.431	222			
Satisfaction with medical process service	Between groups	0.254	4	0.064	0.957	0.432
	Within a group	14.487	218	0.066		
	Total	14.741	222			
Satisfaction with medical results and services	Between groups	0.186	4	0.047	1.081	0.367
	Within a group	9.344	217	0.043		
	Total	9.530	221			
